# Scalable markers for early cognitive decline: Plasma p‐tau217, subjective cognitive concerns, and digital testing: Results from the A4/LEARN studies

**DOI:** 10.1002/alz.71505

**Published:** 2026-05-27

**Authors:** Babak Khorsand, Devin Teichrow, Elham Ghanbarian, Lukai Zheng, S. Ahmad Sajjadi, Crystal M. Glover, Joshua D. Grill, Laura A. Rabin, Ali Ezzati

**Affiliations:** ^1^ Department of Neurology University of California Irvine California USA; ^2^ Brooklyn College and The Graduate Center ‐ CUNY Brooklyn New York USA

**Keywords:** Alzheimer's disease, cognitive decline, digital biomarkers, plasma biomarkers, subjective cognitive concerns

## Abstract

**INTRODUCTION:**

Amyloid positron emission tomography (PET) and cerebrospinal fluid (CSF) biomarkers confirm Alzheimer's disease (AD) pathology but are impractical for large‐scale screening. Plasma phosphorylated tau at threonine 217 (p‐tau217), subjective cognitive concerns, and computerized cognitive testing are non‐invasive, scalable, and feasible to implement in large populations. We assessed their separate and combined predictive value for cognitive decline.

**METHODS:**

We analyzed 1064 cognitively unimpaired adults (ages 65–85 years) from the Anti‐Amyloid Treatment in Asymptomatic Alzheimer's Disease (A4; amyloid‐positive) and Longitudinal Evaluation of Amyloid Risk and Neurodegeneration (LEARN; amyloid‐negative) studies. Baseline assessments included apolipoprotein E (*APOE*) ε4 status, hippocampal volume, amyloid PET, plasma p‐tau217, Cognitive Function Index (CFI), and Cogstate Computerized Battery (CCB). Cognitive impairment was defined as conversion from a Clinical Dementia Rating Global Score (CDR‐GS) of 0 to ≥0.5 over 240 weeks.

**RESULTS:**

During the follow‐up, 34.1% developed cognitive impairment. Higher p‐tau217, higher CFI, and lower CCB were associated with higher odds of converting to CDR‐GS >0 across all cohorts.

**DISCUSSION:**

P‐tau217, CFI, and CCB each independently predict cognitive decline, offering practical, non‐invasive tools for early AD risk stratification and trial enrichment.

## Highlight

- Plasma phosphorylated tau at threonine 217 (p‐tau217), subjective cognitive concerns, and digital cognitive testing each independently predict progression to cognitive impairment in cognitively unimpaired adults.
- Combining these biological, subjective, and digital markers improves prognostic accuracy beyond any single measure.
- Findings support scalable, non‐invasive approaches for early detection and risk stratification in preclinical Alzheimer's disease.
- Results replicate across amyloid‐positive (Anti‐Amyloid Treatment in Asymptomatic Alzheimer's Disease [A4]) and amyloid‐negative (Longitudinal Evaluation of Amyloid Risk and Neurodegeneration [LEARN]) cohorts, extending generalizability.

## 1 BACKGROUND

Alzheimer's disease (AD) is a progressive neurodegenerative disorder characterized by a prolonged preclinical phase in which pathophysiologic processes such as amyloid beta (Aβ) accumulation and tau aggregation occur years before overt symptoms.^1^ Identifying individuals at greatest risk for near‐term cognitive decline during this preclinical stage is critical for timely intervention, efficient trial designs, and, ultimately, identifying ideal candidates for disease‐delaying treatments. Although amyloid positron emission tomography (PET) and cerebrospinal fluid (CSF) biomarkers reliably detect these pathologies, their invasiveness, cost, and limited accessibility restrict their use for routine screening and risk stratification.^2^ Moreover, subjective and functional aspects of early disease manifestation are not captured by PET or CSF biomarkers. As a result, there is increasing interest in accessible, low‐burden measures that could be deployed in primary care, community, and large‐scale research settings.

Prominent among these emerging alternatives are plasma biomarkers, particularly plasma levels of phosphorylated tau at threonine 217 (p‐tau217), which has shown strong associations with AD pathology and clinical progression in multiple cohorts^3^; subjective cognitive concerns, as measured by tools such as the Cognitive Function Index (CFI), which may capture subtle self‐perceived decline before objective deficits are detectable on neuropsychological testing^4^; and Computerized Cognitive Battery (CCB), which provides standardized, language‐independent assessment, with growing evidence of sensitivity to early changes linked to amyloid and tau pathology.^5^ Although each of these markers—biological, subjective, and digital—has been linked individually been to risk of cognitive decline, the extent to which they contribute independent and complementary information within the same framework is not known. Establishing whether these measures provide complementary rather than redundant information in different populations is essential for building efficient, multidomain risk models.

The Anti‐Amyloid Treatment in Asymptomatic Alzheimer's Disease (A4) Study, a large, multinational randomized controlled trial, and its parallel observational cohort, the Longitudinal Evaluation of Amyloid Risk and Neurodegeneration (LEARN) Study, provide a unique framework to address this gap. Together, these studies include a large, diverse sample of clinically unimpaired older adults with longitudinal follow‐up, rigorous cognitive outcome assessment, and detailed biomarker characterization. In this study, we tested the hypothesis that plasma p‐tau217, CFI, and CCB each independently predict progression from normal cognition to symptomatic impairment over 5 years, and that their combination yields superior predictive performance compared with any single measure. We also examined whether these associations differed by baseline Aβ status.

**RESEARCH IN CONTEXT**

1. **Systematic review**: A literature review identified strong evidence supporting plasma phosphorylated tau at threonine 217 (p‐tau217) as a biomarker of Alzheimer's disease pathology and progression, whereas subjective cognitive concerns (CFIs) and computerized cognitive batteries have each been shown to predict decline in separate cohorts. However, few studies have jointly examined their complementary predictive value in preclinical Alzheimer's disease.
2. **Interpretation**: In over 1000 cognitively unimpaired older adults from the Anti‐Amyloid Treatment in Asymptomatic Alzheimer's Disease (A4) and Longitudinal Evaluation of Amyloid Risk and Neurodegeneration [LEARN] studies, higher plasma p‐tau217, greater subjective concerns, and lower computerized cognitive scores independently predicted progression to CDR‐GS ≥ 0.5 during nearly five years of follow‐up. Each domain contributed unique prognostic information, supporting a multidomain framework for early risk detection.
3. **Future directions**: Future research should validate these scalable markers in community populations, assess longitudinal change and composite risk scoring, and integrate them into primary‐care workflows and decentralized trial recruitment strategies.

## 2 METHODS

### 2.1 Study design and participants

We conducted a secondary analysis of data from a randomized clinical trial (A4) and a parallel observational cohort study (LEARN).^6^ The A4 Study was a multicenter clinical trial conducted at 67 sites across the United States, Australia, Japan, and Canada. It aimed to evaluate whether solanezumab could slow cognitive decline in older adults with preclinical Alzheimer's disease—defined as cognitively unimpaired individuals with elevated Aβ levels on PET scans. The LEARN Study enrolled individuals who were screened for A4 but did not meet the amyloid threshold for trial enrollment.

Full eligibility criteria for the A4 trial were previously reported.^6^ In brief, participants were between 65 and 85 years of age, had Mini‐Mental State Examination (MMSE) scores between 25 and 30, a Global Clinical Dementia Rating Score (CDR‐GS) of 0, and Logical Memory II scores of 6–18 based on educational level. All participants were required to have a study partner and undergo Aβ PET imaging. Exclusion criteria included significant comorbid conditions, elevated suicide risk, and certain medication use.

Of 6763 individuals who were prescreened, 4486 (66%) underwent F‐florbetapir PET imaging. A total of 1209 (27%) individuals with high amyloid burden (Aβ positive) were enrolled in the A4 trial and randomized 1:1 to receive either intravenous solanezumab (*n* = 578) or placebo (*n* = 591). From the remainder of screened participants, 538 (12%) individuals with lower amyloid burden (Aβ negative) were enrolled in the LEARN study.

**FIGURE 1:**
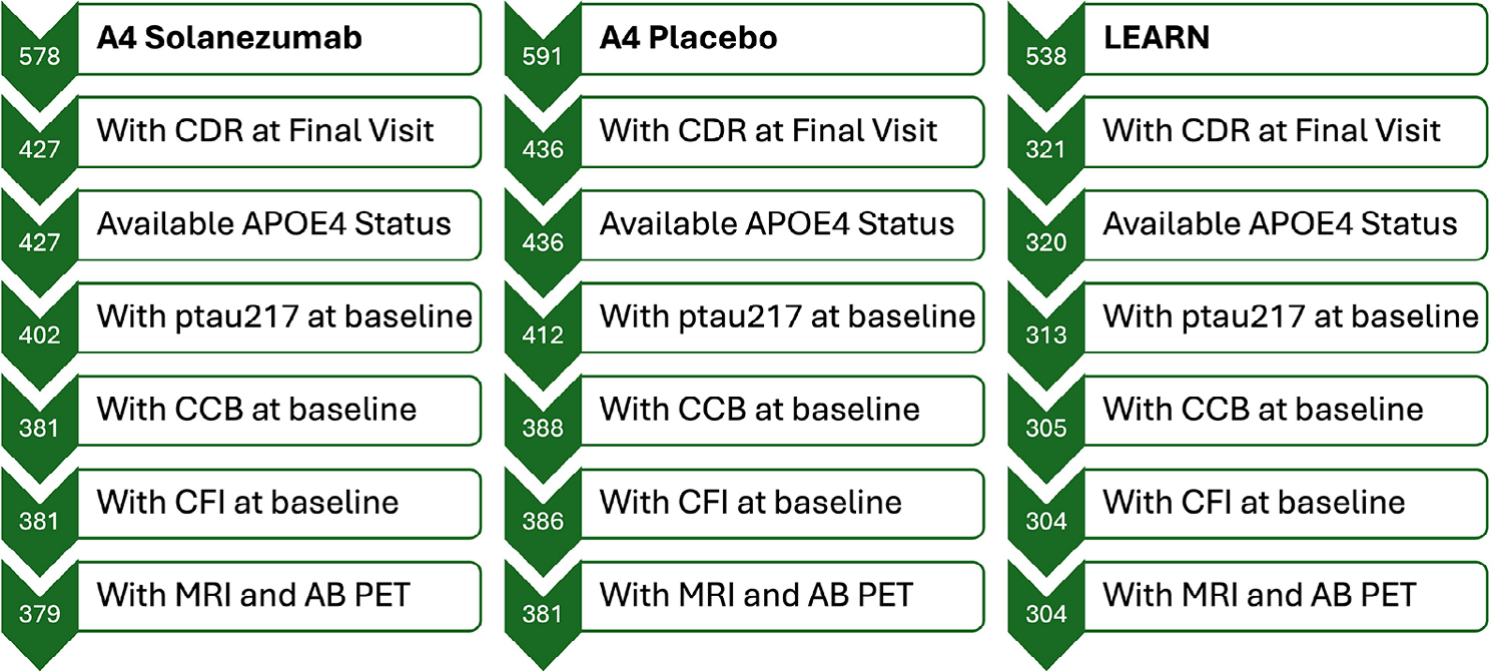
Flowchart of study participants.

For this analysis, we selected participants with available apolipoprotein E (*APOE*) ε4 carrier status, baseline Aβ PET, baseline hippocampus volume, baseline plasma Aβ42/Aβ40 ratio, baseline p‐tau217 measurement, baseline CCB, baseline CFI, and final visit (Week 240) CDR‐GS scores. The final analytic sample included 1064 participants, including 381 in the A4 placebo group, 379 in the A4 solanezumab group, and 304 in the LEARN study. Figure 1 illustrates the flow of participants from initial screening through final analytic sample, including numbers excluded at each step and reasons for exclusion (e.g., missing biomarker or cognitive data).

### 2.2 Study measures

Baseline data were collected at enrollment (Week 0) for all measures, and cognitive status was assessed longitudinally, with final CDR‐GS measured at Week 240. Standardized units were calculated separately within each cohort to account for potential differences in distributional characteristics between groups.

#### 2.2.1 Demographics

Demographic covariates included age, sex, and years of education. Age was analyzed as a continuous variable. Years of education was dichotomized as ≤12 years or >12 years.

#### 2.2.2 Genetic variable

*APOE* ε4 is the strongest genetic risk factor for Alzheimer's disease. In the analysis, *APOE* ε4 status was included as a binary variable, with 0 representing individuals without the ε4 allele and 1 for those carrying at least one ε4 allele.

#### 2.2.3 Imaging biomarkers

Amyloid burden was quantified using 18F‐florbetapir PET imaging. Quantitative measures were derived using the standardized uptake value ratio (SUVR), calculated by comparing tracer uptake in target cortical regions to a reference region. A threshold of SUVR >1.15, or SUVR between 1.1 and 1.15 with concordant visual read, was used to classify as amyloid‐positive.^7^ Those below this threshold without visual read support were considered as amyloid‐negative.

Tau pathology was assessed using 18F‐flortaucipir PET imaging. SUVRs were calculated within a medial temporal lobe (MTL) region of interest.

### 2.3 Volumetric magnetic resonance imaging

High‐resolution structural magnetic resonance imaging (MRI) scans were processed using automated segmentation pipelines in FreeSurfer (version 6.0) and NeuroQuant to obtain hippocampal volumetric measures. To account for variability in head size, hippocampal volumes were corrected using total intracranial volume.^8^

#### 2.3.1 Plasma biomarkers

The p‐tau217 levels were measured from plasma using an electro‐chemiluminescent immunoassay. Automated sample preparation was conducted using the Tecan Fluent workstation, and assays were run on the MSD Sector S Imager 600 MM.^9^

#### 2.3.2 Cogstate computerized battery

The CCB is a set of standardized, computerized cognitive tests designed to assess various domains of cognitive function, including memory, attention, processing speed, and executive function.^10^ The composite score was derived from six tasks: the Behavioral Pattern Separation Object Test (recognition memory, higher scores indicating better performance); the Face Name Associative Memory Exam (associative memory, higher scores indicating better performance); the Detection Test (psychomotor speed, lower scores indicating better performance); the Identification Test (attention, lower scores indicating better performance); the One Card Learning Test (visual learning, higher scores indicating better performance); and the One Back Test (working memory, higher scores indicating better performance). For each task, raw scores were standardized to z‐scores based on the study sample distribution, with directionality harmonized so that higher values consistently reflected better cognitive performance. The composite was then computed as the mean of these standardized scores. Accordingly, the CCB composite should be interpreted as a continuous measure of global cognitive ability, where higher scores indicate better overall functioning.

#### 2.3.3 Subjective cognitive concerns

Subjective cognitive concerns were assessed using the Cognitive Function Index (or CFI), a modified version of the original 14‐item questionnaire, which consists of 15 items assessing perceived cognitive decline in areas such as memory, language, and executive functioning) and functional abilities, relative to one year prior.^11^ Each item is scored as 0, 0.5, or 1, resulting in a total CFI score ranging from 0 to 15, with higher values indicating greater concern about cognitive decline.

#### 2.3.4 Clinical Dementia Rating

The primary cognitive and functional endpoint was assessed using the CDR‐GS. This instrument is based on semi‐structured interviews conducted with both the participant and study partner, allowing evaluation of cognitive performance across six domains: memory, orientation, judgment and problem solving, community involvement, home and leisure activities, and personal care. Each domain is scored separately on a 5‐point scale indicating impairment severity: 0 (none), 0.5 (very mild or questionable), 1 (mild), 2 (moderate), and 3 (severe dementia).^12^ For the present study, cognitive/functional decline was defined as a ≥0.5 increase in the CDR‐GS during the 240‐week follow‐up period.^12^ Participants were followed for the CDR‐GS test at Weeks 48, 120, 168, 204, and 240.

### 2.4 Statistical analysis

Baseline characteristics were summarized descriptively. The primary outcome was incident symptomatic cognitive impairment, defined as progression from a baseline CDR‐GS of 0 to a score of ≥0.5 at any follow‐up visits.^6^ Participants meeting this criterion were classified as Decliners; all others were considered Stable.

Survival analyses evaluated the association of plasma p‐tau217, CFI, and CCB with risk of cognitive impairment. All models were adjusted for age, sex, years of education, and *APOE* ε4 status. Hazard ratios (HRs) with 95% confidence intervals (CIs) were reported. The proportional hazards assumption was tested with Schoenfeld residuals and was not violated. We estimated risk in each cohort (A4 Placebo, A4 Solanezumab, and LEARN) using a sequence of nested models: a base model with covariates only (age, sex, education, *APOE* ε4); base model plus each predictor separately (p‐tau217, CFI, or CCB); base model with pairs of predictors; and a full model including all three predictors simultaneously. Each model estimated the HR and 95% CIs for predictors of time to incident cognitive impairment. We modeled predictors in standard units, so all reported results represent HRs per one standard deviation (SD) increase. Predictors were also modeled in their native units (p‐tau217 as continuous assay values, CFI per 1‐point increase, and CCB as composite score units), which are presented in Table S1.

To visualize differences in time‐to‐event outcomes, we generated Kaplan–Meier survival curves, stratified by quartiles of p‐tau217, CFI, and CCB. Group differences were assessed using the log‐rank test.

In the second analysis incorporating MRI and PET imaging biomarkers, we combined amyloid‐positive participants from the placebo arm of the A4 study with amyloid‐negative participants from the LEARN study and repeated the analyses in this expanded dataset. All models were adjusted for age, sex, and years of education.

Risk was estimated using a series of nested models^1^: a base model including covariates only (age, sex, and education)^2^; the base model plus each predictor entered separately (*APOE* ε4 status, hippocampal volume, Aβ SUVR, plasma p‐tau217, CFI, and CCB)^3^; the base model plus *APOE* ε4 status, hippocampal volume, and Aβ SUVR; and^4^ the base model plus plasma p‐tau217, CFI, and CCB.

Moreover, we developed a tree‐based survival model (random survival forest) as a sensitivity analysis. This approach accommodates censoring and allows flexible, non‐parametric relationships without prespecifying functional forms. Predictors included age, sex, years of education, *APOE* ε4 status, hippocampal volume, Aβ SUVR, plasma p‐tau217, CFI, and CCB. We additionally performed permutation‐based feature importance analyses to quantify the relative contribution of each predictor to model performance.

### 2.5 Data availability

The data utilized in this article are from the publicly available dataset from the A4 trial, which can be accessed and downloaded from the Alzheimer's Clinical Trials Consortium (ACTC).

## 3 RESULTS

### 3.1 Sample characteristics

The analytic sample included 1064 participants 65 to 85 years of age at baseline (mean ± SD: 71.25 ± 4.5 years). On average, participants had 16.69 years of education (SD: 2.7). The cohort consisted of 59% women, and 49.9% of participants were *APOE* ε4 carriers. During the follow‐up period of 240 weeks, 362 participants (33.9%) developed incident cognitive impairment, defined as an increase in CDR‐GS from 0 at baseline to ≥0.5 at any follow‐up visits. The remaining 702 participants (66.1%) remained cognitively stable.

In the entire sample, compared to stable participants, decliners were significantly older at baseline (72.7 vs 70.5, *p* < 0.001), more likely to be male (51.5 vs 35.5, *p* < 0.001), and more frequently *APOE* ε4 carriers (56.2% vs 46.6%, *p* = 0.03). They also had higher Aβ PET SUVR (1.33 vs 1.19, *p* < 0.001), tau PET (1.28 vs 1.16, *p* < 0.001), p‐tau217 (0.31 vs 0.21, *p* < 0.001), CFI (5.2 vs 2.7, *p* < 0.001) and lower plasma Aβ42/Aβ40 ratio (94.38 vs 99.01, *p* = 0.006), hippocampus volume (5.85 vs 6.35, *p* < 0.001), and CCB (−0.16 vs 0.1, *p* < 0.001). Sample characteristics, stratified by Stable and Decliners for each study, are summarized in Table 1.

**TABLE 1:** Baseline demographic, genetic, and biomarker characteristics of participants by cognitive outcome (stable vs decliner) across the A4 placebo, A4 solanezumab, and LEARN cohorts.

	Solanezumab			Placebo			LEARN		
	Stable	Decliner	*p*	Stable	Decliner	*p*	Stable	Decliner	*p*
Samples, *N* (%)	229 (60.1)	152 (39.9)		237 (61.4)	149 (38.6)		240 (78.9)	64 (21.1)	
Age, years (mean ± SD)	70.88 ± 4.1	72.61 ± 4.8	<0.001	70.76 ± 4.1	72.94 ± 5.2	<0.001	69.85 ± 3.8	72.37 ± 5.3	<0.001
Sex, Male, *N* (%)	89 (38.9)	71 (46.7)	0.15	80 (33.8)	76 (51)	0.001	82 (34.2)	41 (64.1)	<0.001
Education, years (mean ± SD)	16.72 ± 2.5	16.45 ± 2.7	0.31	16.59 ± 2.9	16.56 ± 2.9	0.93	16.92 ± 2.5	16.78 ± 2.8	>0.05
BMI (mean ± SD)	27.02 ± 4.9	27.47 ± 5.2	0.39	27.92 ± 5.1	27.29 ± 5.2	0.24	27.65 ± 5.1	28.21 ± 4.1	>0.05
*APOE* ε4 carrier, *N* (%)	135 (59)	100 (65.8)	0.22	146 (61.6)	90 (60.4)	0.89	48 (20)	15 (23.4)	>0.05
Amyloid PET SUVR (mean ± SD)	1.29 ± 0.2	1.4 ± 0.2	<0.001	1.3 ± 0.2	1.39 ± 0.2	<0.001	0.99 ± 0.1	0.99 ± 0.1	>0.05
Amyloid Aβ42/Aβ40 (mean ± SD)	0.11 ± 0.09	0.11 ± 0.09	0.87	0.11 ± 0.07	0.10 ± 0.08	0.61	0.12 ± 0.08	0.11 ± 0.05	0.29
tau PET (mean ± SD)	1.19 ± 0.11	1.29 ± 0.18	<0.001	1.17 ± 0.11	1.31 ± 0.20	<0.001	1.09 ± 0.07	1.12 ± 0.06	0.13
Hippocampal volume (mean ± SD)	6.07 ± 0.6	5.75 ± 0.7	<0.001	6.09 ± 0.6	5.72 ± 0.7	<0.001	6.87 ± 0.6	6.4 ± 0.9	<0.001
Plasma p‐tau217 (mean ± SD)	0.24 ± 0.1	0.35 ± 0.2	<0.001	0.24 ± 0.1	0.32 ± 0.2	<0.001	0.16 ± 0.1	0.18 ± 0.1	<0.001
CFI (mean ± SD)	2.93 ± 2.6	5.78 ± 4.5	<0.001	2.57 ± 2.6	5.1 ± 3.5	<0.001	2.63 ± 2.6	4.28 ± 3.7	<0.001
CCB (mean ± SD)	0.07 ± 0.5	−0.18 ± 0.6	<0.001	0.05 ± 0.5	−0.18 ± 0.6	<0.001	0.18 ± 0.5	−0.07 ± 0.6	<0.001

Abbreviations: *APOE* ε4, allele of apolipoprotein E; BMI, body mass index; CCB, Cogstate Computerized Battery; CFI, Cognitive Function Index; PET, positron emission tomography; SUVR, standardized uptake value ratio; LEARN, Longitudinal Evaluation of Amyloid Risk and Neurodegeneration.

#### 3.1.1 Prediction of incident cognitive impairment (CDR‐GS ≥0.5)

Cox proportional hazards models were used to evaluate the association between baseline predictors and time to incident cognitive impairment, defined as an increase in CDR‐GS from 0 to ≥0.5. Analyses were conducted separately for the A4 Placebo, A4 Solanezumab, and LEARN cohorts. All models were adjusted for age, sex, years of education, and *APOE* ε4 carrier status. As predictors were modeled in different units, HR magnitudes are not directly comparable and so per‐SD HRs were used.

Model 1, which included only demographic covariates and *APOE* 4 status, identified age as a consistent and significant predictor across all three cohorts: A4 Placebo (HR = 1.42; 95% CI, 1.21–1.67; *p* < 0.001), A4 Solanezumab (HR = 1.36; 95% CI, 1.16–1.59; *p* < 0.001), and LEARN (HR = 1.56; 95% CI, 1.25–1.95; *p* < 0.001). Sex was also a significant predictor in A4 Placebo (HR = 1.62; 95% CI, 1.17–2.25; *p* < 0.001) and LEARN (HR = 2.77; 95% CI, 1.63–4.71; *p* < 0.001), with males showing greater risk for progression.

Models 2 through 4 tested the independent contribution of each candidate predictor—plasma p‐tau217, CFI, and CCB—when added to the base model. Across all cohorts, higher baseline p‐tau217 was significantly associated with an increased risk of cognitive impairment: A4‐Placebo (HR = 1.56; 95% CI, 1.37–1.78), A4‐Solanezumab (HR = 1.46; 95% CI, 1.29–1.65), and LEARN (HR = 1.25; 95% CI, 1.05–1.48). Higher CFI scores (indicating more subjective cognitive concerns) were also strongly associated with greater risk: A4‐Placebo (HR = 1.59; 95% CI, 1.42–1.79), A4‐Solanezumab (HR = 1.67; 95% CI, 1.47–1.91), and LEARN (HR = 1.37; 95% CI, 1.12–1.68). Lower CCB scores were also associated with greater risk: A4‐Placebo (HR = 0.76; 95% CI, 0.65–0.91), A4‐Solanezumab (HR = 0.73; 95% CI, 0.62–0.87), and LEARN (HR = 0.68; 95% CI, 0.53–0.87).

Models 5 through 7 incorporated combinations of two candidate predictors, and Model 8 included all three (p‐tau217, CFI, and CCB) simultaneously. The full model (Model 8) demonstrated that each of the three predictors remained independently and significantly associated with time to incident cognitive impairment in all three cohorts (Table 2 for per‐SD HRs and Table S1 for original HRs [effect size]).

**TABLE 2:** Cox proportional hazard models predicting time to the development of incident cognitive impairment (CDR‐GS ≥0.5), with predictors standardized per 1 SD increase.

	Model 1	Model 2	Model 3	Model 4	Model 5	Model 6	Model 7	Model 8
**A4 Placebo**								
Age	1.42^***^ (1.21−1.67)	1.32^***^ (1.12−1.55)	1.34^***^ (1.15−1.57)	1.31^**^ (1.11−1.55)	1.27^**^ (1.09−1.48)	1.22^*^ (1.04−1.45)	1.27^**^ (1.08−1.49)	1.21 ^*^ (1.02−1.42)
Sex, male	1.62^**^ (1.17−2.25)	1.87^***^ (1.34−2.61)	1.47^*^ (1.06−2.06)	1.61^**^ (1.16−2.24)	1.68^**^ (1.19−2.36)	1.88^***^ (1.34−2.62)	1.45^*^ (1.04−2.03)	1.68 ^**^ (1.19−2.36)
Education	1.00 (0.95−1.06)	1.00 (0.95−1.06)	1.00 (0.95−1.07)	1.01 (0.96−1.07)	1.01 (0.95−1.06)	1.01 (0.96−1.07)	1.01 (0.96−1.08)	1.01 (0.96–1.07)
*APOE* ε4	1.11 (0.80–1.55)	1.06 (0.76–1.48)	1.16 (0.83–1.63)	1.12 (0.81–1.56)	1.16 (0.82–1.61)	1.08 (0.77–1.52)	1.16 (0.83–1.62)	1.17 (0.83–1.64)
p‐tau217		1.56^***^ (1.37–1.78)			1.57^***^ (1.38–1.79)	1.54^***^ (1.35–1.76)		1.56^***^ (1.37–1.78)
CFI			1.59^***^ (1.42–1.79)		1.62^***^ (1.43–1.83)		1.56^***^ (1.39–1.76)	1.59^***^ (1.41–1.79)
CCB				0.76^**^ (0.65–0.91)		0.78^**^ (0.66–0.92)	0.82^*^ (0.69–0.96)	0.84^*^ (0.71–0.99)
AIC	2393	2367	2354	2369	2323	2345	2339	2319
Concordance	0.61	0.66	0.68	0.63	0.73	0.69	0.71	0.74
**A4 Solanezumab**								
Age	1.36^***^ (1.16–1.59)	1.24^*^ (1.05–1.46)	1.33^***^ (1.14–1.56)	1.23^*^ (1.04–1.45)	1.22^*^ (1.03–1.44)	1.16^*^ (0.98–1.38)	1.25^**^ (1.06–1.48)	1.17^*^ (0.98–1.39)
Sex, male	1.19 (0.86–1.66)	1.29 (0.93–1.79)	0.98 (0.71–1.37)	1.19 (0.85–1.65)	1.07 (0.77–1.49)	1.24 (0.89–1.73)	1.01 (0.73–1.41)	1.08 (0.78–1.50)
Education	0.96 (0.91–1.03)	0.97 (0.91–1.04)	1.00 (0.94–1.06)	0.99 (0.93–1.06)	1.01 (0.94–1.07)	0.99 (0.93–1.07)	1.01 (0.95–1.08)	1.01 (0.95–1.08)
*APOE* ε4	1.38 (0.98–1.94)	1.25 (0.79–1.77)	1.38 (0.98–1.93)	1.39 (0.99–1.96)	1.31 (0.93–1.84)	1.27 (0.89–1.80)	1.38 (0.98–1.93)	1.31 (0.93–1.85)
p‐tau217		1.46^***^ (1.29–1.65)			1.36^***^ (1.19–1.55)	1.41^***^ (1.24–1.59)		1.34^***^ (1.18–1.52)
CFI			1.67^***^ (1.47–1.91)		1.60^***^ (1.40–1.83)		1.63^***^ (1.42–1.86)	1.57^***^ (1.37–1.80)
CCB				0.73^***^ (0.62–0.87)		0.78^**^ (0.66–0.93)	0.83^*^ (0.70–0.99)	0.86^*^ (0.73–0.99)
AIC	2355	2338	2329	2343	2298	2324	2305	2290
Concordance	0.61	0.64	0.67	0.63	0.71	0.68	0.70	0.72
**LEARN**								
Age	1.56^***^ (1.25–1.95)	1.59^***^ (1.27–1.99)	1.58^***^ (1.26–1.99)	1.42^**^ (1.12–1.80)	1.60^***^ (1.27–2.01)	1.45^**^ (1.14–1.85)	1.44^**^ (1.13–1.84)	1.46^**^ (1.14–1.87)
Sex, male	2.77^***^ (1.63–4.71)	2.68^***^ (1.58–4.54)	2.37^**^ (1.38–4.07)	3.03^***^ (1.78–5.14)	2.30^**^ (1.34–3.96)	2.91^***^ (1.72–4.95)	2.59^***^ (1.51–4.47)	2.52^***^ (1.46–4.34)
Education	0.93 (0.84–1.03)	0.93 (0.84–1.02)	0.94 (0.86–1.04)	0.94 (0.86–1.04)	0.94 (0.86–1.04)	0.94 (0.85–1.04)	0.95 (0.87–1.05)	0.95 (0.87–1.05)
APOE4	1.62 (0.87–3.02)	1.49 (0.79–2.82)	1.58 (0.85–2.93)	1.58 (0.85–2.96)	1.44 (0.76–2.71)	1.50 (0.79–2.83)	1.54 (0.83–2.87)	1.43 (0.76–2.70)
p‐tau217		1.25^*^ (1.05–1.48)			1.21^*^ (1.01–1.45)	1.21^*^ (1.02–1.43)		1.18^*^ (1.00–1.41)
CFI			1.37^**^ (1.12–1.68)		1.34^**^ (1.09–1.65)		1.32^**^ (1.08–1.62)	1.30^*^ (1.06–1.59)
CCB				0.68^**^ (0.53–0.87)		0.69^**^ (0.54–0.89)	0.70^**^ (0.54–0.90)	0.71^**^ (0.54–0.92)
AIC	1376	1374	1361	1370	1360	1369	1357	1356
Concordance	0.69	0.70	0.72	0.71	0.74	0.72	0.74	0.75

***Note***: Hazard ratios (HRs) with 95% confidence intervals (CIs) are shown for each cohort. All models adjust for age, sex, education, and *APOE* ε4 carrier status. Predictors were standardized so HRs represent risk per 1 SD increase, allowing comparison across measures.

Abbreviations: CCB, Cogstate Computerized Battery Significant Codes; CFI, Cognitive Function Index.

*** *p* < 0.001;

** *p* < 0.01;

* *p* < 0.05.

**FIGURE 2:**
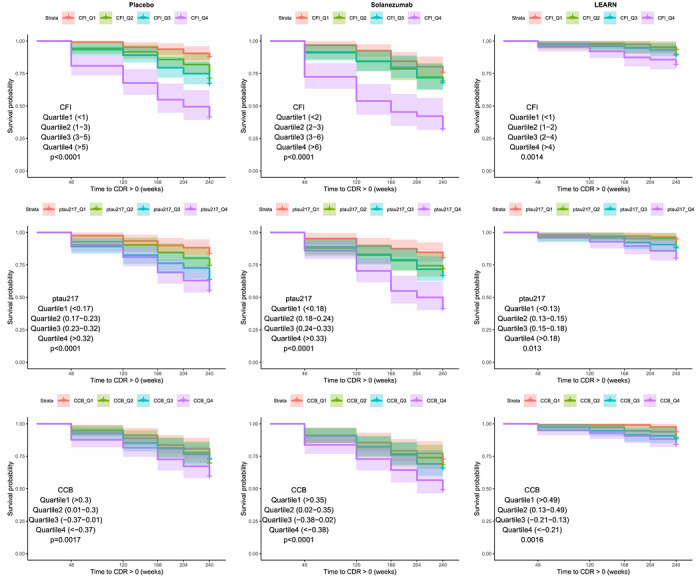
Kaplan–Meier curves for time to cognitive impairment (Clinical Dementia Rating Global Score [CDR‐GS] >0) by quartiles of baseline measures.

Kaplan–Meier survival curves, stratified by quartiles of baseline measures, revealed significant associations between each predictor and time to incident cognitive impairment, as assessed by the log‐rank test (Figure 2). In the A4 Placebo group, higher baseline CFI scores were associated with increased risk of cognitive decline (*χ* ^2^ = 78.1, *p* < 0.001; Figure 2a). Similarly, higher plasma p‐tau217 levels (*χ* ^2^ = 31.1, *p* < 0.001; Figure 2b) and lower CCB scores (*χ^2^* = 15.1, *p* = 0.002; Figure 2c) were significantly associated with faster progression. Comparable results were observed in the Solanezumab arm of the A4 study, with significant associations for CFI (*χ^2^* = 67.6, *p* < 0.001), p‐tau217 (*χ^2^* = 45.7, *p* < 0.001), and CCB (*χ^2^* = 21.7, *p* < 0.001). The LEARN cohort also showed consistent findings, with CFI (*χ^2^* = 12.6, *p *= 0.006), p‐tau217 (*χ^2^* = 13.2, *p* = 0.004), and CCB (χ^2^ = 9.7, *p* = 0.02) each significantly predicting time to cognitive impairment.

In the secondary analysis, amyloid‐positive (placebo arm of A4) and amyloid‐negative (LEARN) participants were combined. All models were adjusted for age, sex, and years of education.

Model 1, which included only demographic covariates (Akaike Information Criterion [AIC] = 2608), identified age as a consistent and significant predictor (HR = 1.09; 95% CI, 1.06–1.12; *p* < 0.001). Sex was also a significant predictor (HR = 1.86; 95% CI, 1.41–2.45; *p* < 0.001), with males showing greater risk for progression. Moreover, participants with less than 12 years of education shows greater risk for progression (HR = 1.53; 95% CI, 1.00–2.35; *p* = 0.05).

Models 2 through 7 tested the independent contribution of each candidate predictor—*APOE* ε4, Aβ SUVR, hippocampus volume, CCB, CFI, and plasma p‐tau217—when added to the base model. *APOE* ε4 carriers shows greater risk for progression (HR = 1.49; 95% CI, 1.13–1.95; *p* = 0.005; AIC = 2602). Higher baseline Aβ SUVR (HR = 6.58; 95% CI, 3.75–11.55; *p* < 0.001; AIC = 2570), CFI (HR = 1.15; 95% CI, 1.11–1.19; *p* < 0.001; AIC = 2565), and p‐tau217 (HR = 33.75; 95% CI, 15.06–75.64; *p* < 0.001; AIC = 2557) were significantly associated with increased risk of cognitive impairment. Lower baseline hippocampus volume (HR = 0.57; 95% CI, 0.46–0.70; *p* < 0.001; AIC = 2581) and CCB (HR = 0.55; 95% CI, 0.42–0.71; *p* < 0.001; AIC = 2582) were also significantly associated with an increased risk of cognitive impairment. Model 8 extended the base model by simultaneously incorporating *APOE* ε4 status, Aβ SUVR, and hippocampal volume (AIC = 2565). Finally, Model 9 extended the base model by simultaneously incorporating CCB, CFI, and plasma p‐tau217 (AIC = 2503). This model, which included our proposed scalable markers, demonstrated substantially better performance than all other models, with a ΔAIC of at least 54 compared with the alternatives (Table S2). We also examined the association of tau PET and plasma Aβ42/Aβ40 ratio with the outcome; however, because these measures were unavailable for a substantial proportion of participants, they were excluded from the final models.

**FIGURE 3:**
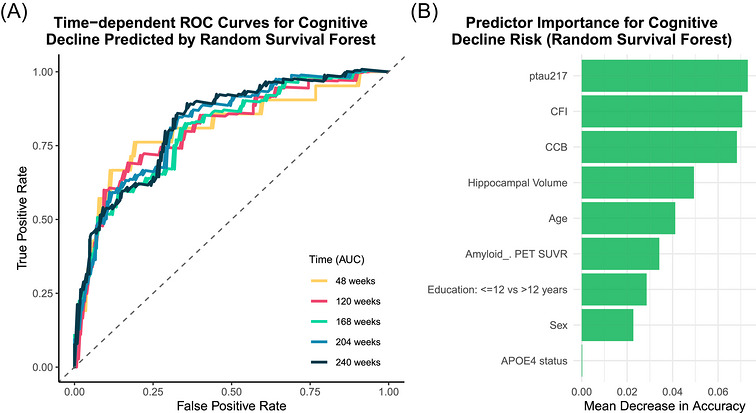
(A) Time‐dependent receiver‐operating characteristic (ROC) curves for cognitive decline predicted by random survival forest. (B) Predictor importance for cognitive decline risk (random survival forest).

In a complementary analysis evaluating predictive performance using random survival forest model, The Random Survival Forest (RSF) All participants provided written informed consent. The current study was determined not to meet the definition of human subjects research. demonstrated good discrimination (C‐index = 0.787) (Figure 3‐A) and yielded a consistent predictor‐importance profile, with p‐tau217, CFI, and CCB among the most informative predictors (Figure 3‐B).

## 4 DISCUSSION

In three well‐characterized cohorts drawn from both a randomized clinical trial (A4) and a parallel observational study (LEARN), we found that plasma p‐tau217, subjective cognitive concerns, and computerized cognitive testing each predicted progression to symptomatic impairment over nearly 5 years of follow‐up. This multidomain framework—integrating biological, subjective, and digital measures—demonstrates that risk for near‐term decline can be quantified using scalable tools accessible outside of specialized research settings. Of note, predictive value was observed in both amyloid‐positive and amyloid‐negative participants, extending utility beyond biomarker‐confirmed AD pathology. Differences in HR magnitudes across predictors reflect unit scaling rather than true differences in prognostic strength, and supplemental per‐SD analyses confirmed that each measure contributed independent and complementary information. Together, these findings advance the field by showing that distinct, low‐burden markers can be combined to improve risk stratification in preclinical populations.

Although CSF and PET biomarkers have long been the gold standard method for antemortem detection of AD neuropathologic change, their invasiveness, cost, and limited availability hinder widespread adoption. In contrast, p‑tau217 offers a scalable, non‑invasive alternative, measurable via simple blood draw on standard assay platforms. This accessibility enables early risk detection in primary care or community health settings lacking the gold standard biomarker infrastructure. Multiple large‑scale studies, including memory clinic cohorts,^13^, ^14^ diverse population samples,^15^ and randomized trials^16^, ^17^ have consistently demonstrated the strong diagnostic and prognostic utility of plasma p‐tau217 across the AD continuum. Our findings add to this evidence: across two large multicenter cohorts with differing amyloid status, p‑tau217 predicted clinical progression over nearly 5 years of follow‑up and multiple CDR assessments. Its predictive power persisted when combined with subjective cognitive concerns and computerized testing, underscoring p‑tau217 as a critical element in multidomain risk models. The fact that p‐tau217 was associated with cognitive decline even in those with negative amyloid PET indicates that some participants with negative amyloid PET may have very early, preclinical AD.

The CFI questionnaire can be completed without a clinician, making it practical for large‑scale screening. Despite its simplicity, the CFI reliably captures early cognitive changes: test–retest reliability is high, and rising CFI scores over time predict subsequent cognitive decline and worsening clinical dementia ratings.^11^, ^18^, ^19^ These associations persist across diverse settings; cross‑cultural validation studies demonstrate that the self‑report version accurately identifies older adults with subjective cognitive decline and is easy to administer^20^, ^21^, ^22^ while digital platforms show CFI scores correlate with daily functional impairments and higher risk of amyloid positivity.^4^ Given its low cost and strong predictive value, the CFI is well suited as a first‑line tool to flag individuals who may benefit from more intensive cognitive testing or biomarker assessment—and our study confirms its predictive value by demonstrating that higher CFI scores were associated with clinical progression in our cohort.

In the secondary analysis combining amyloid‐positive and amyloid‐negative participants, scalable blood‐ and digitally derived markers demonstrated strong and independent associations with incident cognitive impairment. Although established imaging and genetic biomarkers—including Aβ SUVR, hippocampal volume, and *APOE* ε4 status—were significant predictors, the model incorporating CCB, CFI, and plasma p‐tau217 showed markedly superior performance, reflected by a substantial improvement in model fit (ΔAIC ≥54). Notably, plasma p‐tau217 exhibited a particularly strong association with progression risk, consistent with prior literature supporting its sensitivity to early AD‐related pathophysiology.^23^, ^24^, ^25^ The robust performance of CFI and CCB further underscores the value of combining plasma biomarkers with scalable cognitive and functional assessments. Together, these findings suggest that accessible, low‐burden markers may provide prognostic information comparable to—or exceeding—that of traditional imaging biomarkers, supporting their potential utility in large‐scale screening and risk stratification frameworks.

Digital platforms are making cognitive assessment faster and more accessible for preclinical AD. A study by Tideman et al. demonstrated that combining plasma biomarkers, including p‐tau217, with brief digital cognitive measures improves early detection of AD‐related cognitive decline and enhances risk stratification in preclinical populations,^26^ and our findings align with and extend this literature. Studies in preclinical AD cohorts show that elevated p‑tau217 together with poorer performance on remote or brief digital memory tests identifies cognitively unimpaired individuals at highest risk of subsequent decline and accelerated tau accumulation, and that dual enrichment on these markers can reduce required sample sizes for prevention trials by roughly three‑quarters compared with amyloid‑positive samples alone.^27^, ^28^ Complementary cohort data demonstrate that plasma p‑tau217 is more strongly and specifically associated with episodic memory impairment than other plasma p‑tau isoforms, providing a biological rationale for pairing this biomarker with memory‑focused digital tasks.^29^ Card‑based tasks, self‑rated memory tests, voice‑driven questionnaires, and even behavioral markers (gait, driving patterns, speech) can all be administered in minutes via tablets or smartphones and scored automatically. Evidence shows that brief (<10 min) digital cognitive tests can separate cognitively unimpaired, amyloid‑negative adults from amyloid‑positive mild cognitive impairment and distinguish biomarker‑negative mild cognitive impairment from prodromal AD, whereas self‑rated memory and executive tests and voice‑interaction screens each demonstrate high accuracy for detecting mild cognitive impairment and early dementia.^30^ Other studies also indicate that CCB capture early deficits, correlate with biomarker‐defined risk, and can be used for capturing cognitive decline in AD.^31^, ^32^ Our data show that a brief computerized cognitive test predicted progression to CDR‐GS ≥ 0.5 over 5 years, and this association held across treatment, placebo, and observational arms.

A key strength of this study is the simultaneous modeling of three accessible but distinct indicators of AD risk: plasma p‑tau217, subjective cognitive concerns via the CFI, and computerized cognitive tests. Only a handful of studies have evaluated biological, subjective, and digital cognitive measures in the same framework; most examine these tests in isolation or in pairs. In one study, digital card‑based tasks and remote memory tests correlated with plasma p‐tau and combining them with p‑tau181 yielded near‑perfect discrimination between AD and control groups.^33^ Persistent subjective cognitive concerns amplify the dementia risk associated with glial fibrillary acidic protein, with odds ratios exceeding 7—substantially higher than those observed for either the biomarker or subjective complaints alone.^34^ Even after accounting for subjective cognitive decline, plasma p‐tau217 and related blood markers remain significant predictors of preclinical cognitive deterioration in cognitively unimpaired elders.^35^ Plasma p‐tau217 accurately predicts amyloid PET positivity in cognitively unimpaired individuals.^9^ Together, these findings suggest that molecular pathology, digital biomarkers, and subjective experience each capture unique aspects of preclinical AD. Our study demonstrates that incorporating all three yields a more robust yet scalable risk model, providing a comprehensive picture of disease risk that can enhance early detection and intervention.

Blood‑based prescreening can materially economize trial recruitment by reducing the number of negative amyloid PET scans; modeling suggests that triaging candidates with plasma p‑tau assays can cut screen failures and associated costs by roughly 40%–50%.^36^ Sequential strategies, such as using plasma p‑tau217 to identify high‑risk individuals and then confirming with tau PET—retain prognostic power while further improving efficiency.^37^ The predictors examined here, including plasma p‑tau217, subjective cognitive concerns, and digital cognitive tests, are non‑invasive, inexpensive, and do not require specialist equipment. These characteristics make them attractive for broad adoption in primary care screening, digital cognitive health monitoring, population registries, and decentralized clinical trials.

*APOE* ε4 is a well‐established genetic risk factor for AD, particularly for the development and earlier onset of amyloid pathology.^38^ In our analyses, *APOE* ε4 showed a modest association with progression in pooled models (Table S2), but its effect was attenuated and not consistently significant in cohort‐stratified models (Table 2). Several factors may explain this pattern. First, the A4 cohort was already enriched for elevated amyloid, which restricts variability in upstream amyloid‐related risk and may reduce the observable incremental effect of *APOE* ε4 on near‐term progression. Second, *APOE* ε4 is known to exert its strongest influence on amyloid accumulation and lifetime disease risk; in biomarker‐characterized cohorts with established pathology, short‐term clinical progression may be more directly driven by downstream tau‐related and neurodegenerative processes. Consistent with this framework, plasma p‐tau217 and hippocampal volume demonstrated stronger and more consistent associations with incident impairment in our models. Thus, within an amyloid‐enriched and biomarker‐characterized population, dynamic molecular and neurodegenerative markers provide greater prognostic resolution for near‐term clinical decline.

This work has several limitations. This study is a secondary analysis of A4/LEARN data with exploratory model choices; hence, findings should be considered hypothesis‑generating rather than definitive. The highly educated, primarily non‑Hispanic White volunteers in A4/LEARN limit generalizability to community populations and may understate access and other barriers to routine care. Missing data and attrition could bias estimates, and our outcome (conversion from CDR‐GS 0 to ≥0.5) may miss more subtle preclinical changes. Finally, each predictor has measurement constraints: the CBB can be affected by practice effects and device differences; the CFI could partially reflect mood or informant biases; and plasma p‑tau217 assays vary across platforms. We did not examine longitudinal changes in these measures, so prospective validation is needed.

In summary, across the three A4/LEARN cohorts, baseline plasma p‑tau217, subjective cognitive concerns, and a brief computerized cognitive battery each independently predicted conversion from CDR‐GS 0 to ≥0.5. Fully adjusted models showed that these markers offered complementary prognostic information, and survival curves separated consistently, indicating that inexpensive, scalable tools can flag elevated risk several years before clinical diagnosis. This supports a blood‑first triage approach augmented by digital cognition and structured subjective report for early detection, counseling, and trial enrichment. Future work should validate these findings in more diverse community cohorts, assess competing risks and missing‑data mechanisms, examine device and platform effects, and determine whether longitudinal changes and composite risk scores can be integrated into primary‑care workflows and decentralized trials.

## CONFLICT OF INTEREST STATEMENT

All authors declare that they have no commercial or financial relationships that could be construed as a potential conflict of interest. The study received no commercial funding. Dr. Ali Ezzati reports research support from the National Institute on Aging (NIA) and the Alzheimer's Association, as stated in the Acknowledgments. All other authors report no competing interests related to the content of this manuscript. Author disclosures are available in the Supporting Information.

## CONSENT STATEMENT

All participants provided written informed consent. The current study was determined not to meet the definition of human subjects research.

## Supporting information



Supporting Information

Supporting Information

Supporting Information

## References

[1] Rafii MS , Aisen PS . Detection and treatment of Alzheimer's disease in its preclinical stage. Nat Aging. 2023;3(5):520‐531. 37202518 10.1038/s43587-023-00410-4 PMC11110912

[2] Yu X , Shao K , Wan Ke , et al. Progress in blood biomarkers of subjective cognitive decline in preclinical Alzheimer's disease. Chin Med J. 2023;136(5):505‐521. 36914945 10.1097/CM9.0000000000002566 PMC10106168

[3] Selma‐Gonzalez J , Rubio‐Guerra S , García‐Castro J , et al. Association of plasma phosphorylated tau 217 with clinical deterioration across Alzheimer disease stages. Neurology. 2025;105(1):e213769. 40472304 10.1212/WNL.0000000000213769

[4] Demirsoy I , Ghanbarian E , Khorsand B , et al. Association of item‐level responses to cognitive function index with tau pathology and hippocampal volume in the A4 study. Alzheimers Dement (Amst). 2025;17(2):e70128. 40539003 10.1002/dad2.70128 PMC12177208

[5] Öhman F , Hassenstab J , Berron D , Schöll M , Papp KV . Current advances in digital cognitive assessment for preclinical Alzheimer's disease. Alzheimers Dement (Amst). 2021;13(1):e12217. 34295959 10.1002/dad2.12217 PMC8290833

[6] Sperling RA , Donohue MC , Raman R , et al. Trial of solanezumab in preclinical Alzheimer's disease. N Engl J Med; 2023;389(12):1096‐1107. 37458272 10.1056/NEJMoa2305032 PMC10559996

[7] Winston CN , Langford O , Levin N , et al. Evaluation of blood‐based plasma biomarkers as potential markers of amyloid burden in preclinical Alzheimer's disease. J Alzheimers Dis. 2023;92(1):95‐107. 36710683 10.3233/JAD-221118 PMC11191492

[8] Wang J , Hill‐Jarrett T , Buto P , et al. Comparison of approaches to control for intracranial volume in research on the association of brain volumes with cognitive outcomes. Hum Brain Mapp. 2024;45(4):e26633. 38433682 10.1002/hbm.26633 PMC10910271

[9] Rissman RA , Donohue MC , Langford O , et al. Longitudinal phospho‐tau217 predicts amyloid positron emission tomography in asymptomatic Alzheimer's disease. J Prev Alzheimers Dis. 2024;11(4):823‐830. 39044490 10.14283/jpad.2024.134 PMC11266279

[10] Fratti S , Bowden SC , Cook MJ . Reliability and validity of the CogState computerized battery in patients with seizure disorders and healthy young adults: comparison with standard neuropsychological tests. Clin Neuropsychol. 2017;31(3):569‐586. 27852143 10.1080/13854046.2016.1256435

[11] Amariglio RE , Donohue MC , Marshall GA , et al. Tracking early decline in cognitive function in older individuals at risk for Alzheimer disease dementia: the Alzheimer's disease cooperative study cognitive function instrument. JAMA Neurol. 2015;72(4):446‐454. 25706191 10.1001/jamaneurol.2014.3375 PMC4397164

[12] Rentz DM , Rosenberg PB , Sperling RA , et al. Characterizing clinical progression in cognitively unimpaired older individuals with brain amyloid: results from the A4 study. J Prev Alzheimers Dis. 2024;11(4):814‐822. 39044489 10.14283/jpad.2024.123 PMC11266445

[13] Bucci M , Almkvist O , Bluma M , et al. Profiling plasma biomarkers, particularly pTau217 and pTau217/Aβ42, and their relation to cognition in memory clinic patients. J Neurochem. 2025;169(8):e70182. 40785395 10.1111/jnc.70182 PMC12336780

[14] Feizpour A , Doecke JD , Doré V , et al. Detection and staging of Alzheimer's disease by plasma pTau217 on a high throughput immunoassay platform. EBioMedicine. 2024;109:105405. 39437657 10.1016/j.ebiom.2024.105405 PMC11536028

[15] Shin D , Jang H , Yoo H , et al. Potential utility of plasma pTau217 for assessing amyloid and tau biomarker profiles. Eur J Nucl Med Mol Imaging. 2025;53(1):518‐530. 40768095 10.1007/s00259-025-07457-y PMC12660329

[16] Pontecorvo MJ , Lu M , Burnham SC , et al. Association of donanemab treatment with exploratory plasma biomarkers in early symptomatic Alzheimer disease: a secondary analysis of the TRAILBLAZER‐ALZ randomized clinical trial. JAMA Neurol. 2022;79(12):1250‐1259. 36251300 10.1001/jamaneurol.2022.3392 PMC9577883

[17] Devanarayan V , Doherty T , Charil A , et al. Plasma pTau217 predicts continuous brain amyloid levels in preclinical and early Alzheimer's disease. Alzheimers Dement. 2024;20(8):5617‐5628. 38940656 10.1002/alz.14073 PMC11350129

[18] Li C , Neugroschl J , Luo X , et al. The utility of the cognitive function instrument (CFI) to detect cognitive decline in non‐demented older adults. J Alzheimers Dis. 2017;60(2):427‐437. 28854503 10.3233/JAD-161294 PMC6417419

[19] Khorsand B , Ghanbarian E , Rabin LA , Sajjadi SA , Ezzati A . Incremental value of plasma biomarkers in predicting clinical decline among cognitively unimpaired older adults: results from the A4 trial. Alzheimers Dement (Amst). 2026;18(2):e70321. 41948540 10.1002/dad2.70321 PMC13052122

[20] Studart‐Neto A , Moraes NC , Spera RR , et al. Translation, cross‐cultural adaptation, and validity of the Brazilian version of the cognitive function instrument. Dement Neuropsychol. 2022;16:79‐88. 35719263 10.1590/1980-5764-DN-2021-0057 PMC9170254

[21] Chipi E , Frattini G , Eusebi P , et al. The Italian version of cognitive function instrument (CFI): reliability and validity in a cohort of healthy elderly. Neurolog Sci. 2018;39(1):111‐118. 10.1007/s10072-017-3150-z 29063452

[22] So Y , Hahm J , Lee S‐Y , Kim J‐H , Moon K . Development of the subjective cognitive function decline scale for middle‐aged Koreans. Aging Ment Health. 2025;29(5):906‐914. 39703068 10.1080/13607863.2024.2442598

[23] Huber H , Arranz J , Arslan B , et al. Plasma p‐tau217 as a biomarker of Alzheimer's disease pathology in individuals with down syndrome. Nat Commun. 2025;16(1):9900. 41213934 10.1038/s41467-025-65882-x PMC12603275

[24] Moon H , Chen X . Plasma p‐tau217 predicting concurrent Aβ and longitudinal tau across the brain. Alzheimers Dement. 2025;21:e109007.

[25] Yakoub Y , Gonzalez‐Ortiz F , Ashton NJ , et al. Plasma p‐tau217 identifies cognitively normal older adults who will develop cognitive impairment in a 10‐year window. Alzheimers Dement. 2025;21(2):e14537. 40008832 10.1002/alz.14537 PMC11863240

[26] Tideman P , Karlsson L , Strandberg O , et al. Primary care detection of Alzheimer's disease using a self‐administered digital cognitive test and blood biomarkers. Nat Med. 2025;31(12):4131‐4139. 40954312 10.1038/s41591-025-03965-4 PMC12705462

[27] Begde A , Yang Y , Raymont V , Rowe JB , Palejev D , Sinha D , et al. Digital cognition plus plasma p‐Tau217 and Aβ42/40 powerfully predict Alzheimer's progression. Alzheimers Dement (Amst). 2026;18(1):e70281. 41767154 10.1002/dad2.70281 PMC12949451

[28] Vanderlip CR , Gillen DL , Grill JD , Stark CE . Digital memory assessments and plasma pTau217 enable efficient preclinical Alzheimer's trials. J Prev Alzheimers Dis. 2026;13(4):100503. 41653883 10.1016/j.tjpad.2026.100503 PMC12907085

[29] Vanderlip CR , Stark CE . Integrating plasma p‐tau217 and digital cognitive assessments for early detection in Alzheimer's disease. Alzheimers Dement. 2025;21(6):e70355. 40491259 10.1002/alz.70355 PMC12149436

[30] Alden EC , Pudumjee SB , Lundt ES , et al. Diagnostic accuracy of the Cogstate Brief Battery for prevalent MCI and prodromal AD (MCI A+ T+) in a population‐based sample. Alzheimers Dement. 2021;17(4):584‐594. 33650308 10.1002/alz.12219 PMC8371696

[31] Donohue MC , Sperling RA , Salmon DP , et al. The preclinical Alzheimer cognitive composite: measuring amyloid‐related decline. JAMA Neurol. 2014;71(8):961‐970. 24886908 10.1001/jamaneurol.2014.803 PMC4439182

[32] Jutten RJ , Ho EH , Karpouzian‐Rogers T , et al. Computerized cognitive testing to capture cognitive decline in Alzheimer's disease: longitudinal findings from the ARMADA study. Alzheimers Dement (Amst). 2025;17(1):e70046. 39811701 10.1002/dad2.70046 PMC11730193

[33] Toniolo S , Zhao S , Scholcz A , et al. Relationship of plasma biomarkers to digital cognitive tests in Alzheimer's disease. Alzheimers Dement (Amst). 2024;16(2):e12590. 38623387 10.1002/dad2.12590 PMC11016819

[34] Perna L , Stocker H , Burow L , et al. Subjective cognitive complaints and blood biomarkers of neurodegenerative diseases: a longitudinal cohort study. Alzheimers Res Ther. 2023;15(1):198. 37951931 10.1186/s13195-023-01341-3 PMC10638700

[35] Cullen NC , Leuzy A , Janelidze S , et al. Plasma biomarkers of Alzheimer's disease improve prediction of cognitive decline in cognitively unimpaired elderly populations. Nat Commun. 2021;12(1):3555. 34117234 10.1038/s41467-021-23746-0 PMC8196018

[36] Zeng X , Chen Y , Sehrawat A , et al. Alzheimer blood biomarkers: practical guidelines for study design, sample collection, processing, biobanking, measurement and result reporting. Mol Neurodegener. 2024;19(1):40. 38750570 10.1186/s13024-024-00711-1 PMC11095038

[37] Ossenkoppele R , Salvadó G , Janelidze S , et al. Plasma p‐tau217 and tau‐PET predict future cognitive decline among cognitively unimpaired individuals: implications for clinical trials. Nat Agin. 2025;5(5):883‐896. 10.1038/s43587-025-00835-z PMC12092243 40155777

[38] Belaidi AA , Bush AI , Ayton S . Apolipoprotein E in Alzheimer's disease: molecular insights and therapeutic opportunities. Mol Neurodegener. 2025;20(1):47. 40275327 10.1186/s13024-025-00843-y PMC12023563

