# Supplementary material for: Scalable markers for early cognitive decline: Plasma p‐tau217, subjective cognitive concerns, and digital testing: Results from the A4/LEARN studies
Source: Alzheimers Dement. 2026 May 27;22(5):e71505. doi: 10.1002/alz.71505 (PMC13240426; doi:10.1002/alz.71505)
Supplement: Supplementary file 1 — Supporting Information [file ALZ-22-e71505-s002.docx]

**Supplementary Table 1.** Cox proportional hazards models predicting time to incident cognitive impairment (CDR-GS ≥ 0.5), with predictors modeled in their native measurement units.

|  | | **Model 1** | | **Model 2** | | **Model 3** | | **Model 4** | | **Model 5** | | **Model 6** | | **Model 7** | | | **Model 8** | | |  |
| --- | --- | --- | --- | --- | --- | --- | --- | --- | --- | --- | --- | --- | --- | --- | --- | --- | --- | --- | --- | --- |
| **A4 Placebo** | | | | | | | | | | | | | | | | | | |  |  |
| **Age** | 1.08 *******  (1.04-1.12) | | 1.06 *******  (1.03-1.09) | | 1.07 *******  (1.03-1.10) | | 1.06 ******  (1.02-1.09) | | 1.05 ******  (1.02-1.09) | | 1.04 *****  (1.01-1.08) | | 1.05 ******  (1.02-1.09) | | 1.04 *****  (1.01-1.08) | | |  |  |  |
| **Sex, Male** | 1.62 ******  (1.17-2.25) | | 1.87 *******  (1.34-2.61) | | 1.47 *****  (1.06-2.06) | | 1.61 ******  (1.16-2.24) | | 1.68 ******  (1.19-2.36) | | 1.88 *******  (1.34-2.62) | | 1.45 *****  (1.04-2.03) | | 1.68 ******  (1.19-2.36) | | |  |  |  |
| **Education** | 1.00  (0.95-1.06) | | 1.00  (0.95-1.06) | | 1.00  (0.95-1.07) | | 1.01  (0.96-1.07) | | 1.01  (0.95-1.06) | | 1.01  (0.96-1.07) | | 1.01  (0.96-1.08) | | 1.01  (0.96-1.07) | | |  |  |  |
| **APOE4** | 1.11  (0.80-1.55) | | 1.06  (0.76-1.48) | | 1.16  (0.83-1.63) | | 1.12  (0.81-1.56) | | 1.16  (0.82-1.61) | | 1.08  (0.77-1.52) | | 1.16  (0.83-1.62) | | 1.17  (0.83-1.64) | | |  |  |  |
| **p-tau217** |  | | 24.71 *******  (9.55-63.94) | |  | |  | | 26.79 *******  (10.49-68.41) | | 22.63 *******  (8.69-58.95) | |  | | 25.09 *******  (9.75-64.61) | | |  |  |  |
| **CFI** |  | |  | | 1.16 *******  (1.11-1.19) | |  | | 1.16 *******  (1.12-1.21) | |  | | 1.15 *******  (1.11-1.19) | | 1.15 *******  (1.11-1.20) | | |  |  |  |
| **CCB** |  | |  | |  | | 0.60 ******  (0.44-0.82) | |  | | 0.63 ******  (0.46-0.86) | | 0.68 *****  (0.49-0.93) | | 0.72 *****  (0.52-0.98) | | |  |  |  |
| **AIC** | 2393 | | 2367 | | 2354 | | 2369 | | 2323 | | 2345 | | 2339 | | 2319 | | |  |  |  |
| **Concordance** | 0.61 | | 0.66 | | 0.68 | | 0.63 | | 0.73 | | 0.69 | | 0.71 | | 0.74 | | |  |  |  |
| **A4 Solanezumab** | | | | | | | | | | | | | | | | | | |  |  |
| **Age** | | 1.07 *******  (1.03-1.11) | | 1.05 *****  (1.01-1.09) | | 1.07 *******  (1.03-1.10) | | 1.05 *****  (1.01-1.09) | | 1.04 *****  (1.01-1.08) | | 1.03 *****  (1.00-1.07) | | 1.05 ******  (1.01-1.09) | | 1.04 *****  (1.00-1.08) | | | | |
| **Sex, Male** | | 1.19  (0.86-1.66) | | 1.29  (0.93-1.79) | | 0.98  (0.71-1.37) | | 1.19  (0.85-1.65) | | 1.07  (0.77-1.49) | | 1.24  (0.89-1.73) | | 1.01  (0.73-1.41) | | 1.08  (0.78-1.50) | | | | |
| **Education** | | 0.96  (0.91-1.03) | | 0.97  (0.91-1.04) | | 1.00  (0.94-1.06) | | 0.99  (0.93-1.06) | | 1.01  (0.94-1.07) | | 0.99  (0.93-1.07) | | 1.01  (0.95-1.08) | | 1.01  (0.95-1.08) | | | | |
| **APOE4** | | 1.38  (0.98-1.94) | | 1.25  (0.79-1.77) | | 1.38  (0.98-1.93) | | 1.39  (0.99-1.96) | | 1.31  (0.93-1.84) | | 1.27  (0.89-1.80) | | 1.38  (0.98-1.93) | | 1.31  (0.93-1.85) | | | | |
| **p-tau217** | |  | | 9.81 *******  (4.69-20.46) | |  | |  | | 6.38 *******  (2.93-13.91) | | 7.81 *******  (3.74-16.31) | |  | | 5.81 *******  (2.68-12.59) | | | | |
| **CFI** | |  | |  | | 1.15 *******  (1.11-1.19) | |  | | 1.13 *******  (1.09-1.17) | |  | | 1.14 *******  (1.10-1.18) | | 1.13 *******  (1.09-1.17) | | | | |
| **CCB** | |  | |  | |  | | 0.55 *******  (0.40-0.76) | |  | | 0.63 ******  (0.45-0.86) | | 0.71 *****  (0.51-0.97) | | 0.75 *****  (0.55-1.03) | | | | |
| **AIC** | | 2355 | | 2338 | | 2329 | | 2343 | | 2298 | | 2324 | | 2305 | | 2290 | | | | |
| **Concordance** | | 0.61 | | 0.64 | | 0.67 | | 0.63 | | 0.71 | | 0.68 | | 0.70 | | 0.72 | | | | |
| **LEARN** | | | | | | | | | | | | | | | | | | | |  |
| **Age** | | 1.11 *******  (1.05-1.17) | | 1.11 *******  (1.06-1.18) | | 1.11 *******  (1.06-1.17) | | 1.09 ******  (1.03-1.15) | | 1.12 *******  (1.06-1.18) | | 1.09 ******  (1.03-1.16) | | 1.09 ******  (1.03-1.15) | | 1.09 ******  (1.03-1.16) | | | | |
| **Sex, Male** | | 2.77 *******  (1.63-4.71) | | 2.68 *******  (1.58-4.54) | | 2.37 ******  (1.38-4.07) | | 3.03 *******  (1.78-5.14) | | 2.30 ******  (1.34-3.96) | | 2.91 *******  (1.72-4.95) | | 2.59 *******  (1.51-4.47) | | 2.52 *******  (1.46-4.34) | | | | |
| **Education** | | 0.93  (0.84-1.03) | | 0.93  (0.84-1.02) | | 0.94  (0.86-1.04) | | 0.94  (0.86-1.04) | | 0.94  (0.86-1.04) | | 0.94  (0.85-1.04) | | 0.95  (0.87-1.05) | | 0.95  (0.87-1.05) | | | | |
| **APOE4** | | 1.62  (0.87-3.02) | | 1.49  (0.79-2.82) | | 1.58  (0.85-2.93) | | 1.58  (0.85-2.96) | | 1.44  (0.76-2.71) | | 1.50  (0.79-2.83) | | 1.54  (0.83-2.87) | | 1.43  (0.76-2.70) | | | | |
| **p-tau217** | |  | | 39.91 *****  (2.33-68.45) | |  | |  | | 23.58 *****  (1.16-48.12) | | 22.89 *****  (1.31-39.99) | |  | | 15.91 **.**  (0.80-31.81) | | | | |
| **CFI** | |  | |  | | 1.11 ******  (1.04-1.19) | |  | | 1.10 ******  (1.03-1.18) | |  | | 1.10 ******  (1.03-1.18) | | 1.09 *****  (1.02-1.17) | | | | |
| **CCB** | |  | |  | |  | | 0.46 ******  (0.28-0.75) | |  | | 0.48 ******  (0.29-0.79) | | 0.49 ******  (0.29-0.81) | | 0.50 ******  (0.30-0.84) | | | | |
| **AIC** | | 1376 | | 1374 | | 1361 | | 1370 | | 1360 | | 1369 | | 1357 | | 1356 | | | | |
| **Concordance** | | 0.69 | | 0.70 | | 0.72 | | 0.71 | | 0.74 | | 0.72 | | 0.74 | | 0.75 | | | | |

Notes: Values are hazard ratios (HR) with 95% confidence intervals (CI).

Abbreviations: CFI=Cognitive Function Index; CCB=Cogstate Computerized Battery

Significant Codes: *** p<0.001; ** p<0.01; * p<0.05.
