# Supplementary material for: Scalable markers for early cognitive decline: Plasma p‐tau217, subjective cognitive concerns, and digital testing: Results from the A4/LEARN studies
Source: Alzheimers Dement. 2026 May 27;22(5):e71505. doi: 10.1002/alz.71505 (PMC13240426; doi:10.1002/alz.71505)
Supplement: Supplementary file 2 — Supporting Information [file ALZ-22-e71505-s003.docx]

**Supplementary Table 2** Cox Proportional Hazard Models Predicting Time to the Development of Incident Cognitive Impairment (CDR-GS ≥0.5)

|  | **Base** | **+APOE4** | **+AMYL** | **+HIP** | **+CCB** | **+CFI** | **+Ptau217** | **Base + APOE4 + AMY + HIP** | | | **Base + Ptau217 + CFI + CCB** | | |
| --- | --- | --- | --- | --- | --- | --- | --- | --- | --- | --- | --- | --- | --- |
| **Age** | 1.09  (1.06-1.12)  1.49  <0.001 | 1.10  (1.07-1.13)  1.52  <0.001 | 1.07  (1.04-1.11)  1.38  <0.001 | 1.05  (1.02-1.09)  1.26  0.002 | 1.07  (1.04-1.10)  1.34  <0.001 | 1.08  (1.05-1.11)  1.43  <0.001 | 1.08  (1.05-1.11)  1.39  <0.001 | 1.05  (1.02-  1.09)  1.26  0.004 | | | 1.05  (1.02-  1.08)  1.26  <0.001 | | |
| **Sex, Male** | 1.86  (1.41-2.45)  1.87  <0.001 | 1.85  (1.40-2.43)  1.86  <0.001 | 1.92  (1.46-2.54)  1.93  <0.001 | 1.40  (1.04-1.87)  1.42  0.03 | 1.93  (1.46-2.54)  1.93  <0.001 | 1.61  (1.21-2.14)  1.61  0.001 | 2.06  (1.56-2.72)  2.08  <0.001 | 1.60  (1.19-  2.16)  1.62  0.004 | | | 1.84  (1.38-  2.45)  1.84  <0.001 | | |
| **Education,**  **Highschool or less** | 1.53  (1.00-2.35)  0.95  0.05 | 1.52  (0.99-2.33)  0.94  0.05 | 1.29  (0.84-1.98)  0.98  0.24 | 1.50  (0.98-2.30)  0.94  0.06 | 1.36  (0.89-2.09)  0.97  0.15 | 1.35  (0.88-2.06)  0.98  0.17 | 1.39  (0.90-2.13)  0.96  0.13 | 1.34  (0.88-  2.07)  0.97  0.21 | | | 1.10  (0.72-  1.70)  1.00  0.65 | | |
|  |  | **APOE4** | **AMYL** | **HIP** | **CCB** | **CFI** | **Ptau217** | **APOE4** | **AMYL** | **HIP** | **CCB** | **CFI** | **Ptau217** |
|  |  | 1.49  (1.13-1.95)  1.52  0.005 | 6.58  (3.75-11.55)  1.53  <0.001 | 0.57  (0.46-0.70)  0.62  <0.001 | 0.55  (0.42-0.71)  0.72  <0.001 | 1.15 (1.11-1.19)  1.54  <0.001 | 33.75  (15.06-75.64)  1.55  <0.001 | 0.95  (0.69-1.29)  0.94  0.72 | 4.45  (2.26-8.75)  1.40  <0.001 | 0.70  (0.55-0.88)  0.75  0.004 | 0.66  (0.51-0.87)  0.79  0.003 | 1.14  (1.10-1.18)  1.50  <0.001 | 28.19 (12.44-63.93)  1.51  <0.001 |
| **AIC** | 2608 | 2602 | 2570 | 2581 | 2582 | 2565 | 2557 | 2565 | | | 2503 | | |
| **Concordance** | 0.66 | 0.68 | 0.71 | 0.70 | 0.70 | 0.72 | 0.73 | 0.73 | | | 0.77 | | |

**Note.** The first value in each cell represents the hazard ratio (HR; the effect size) with its 95% confidence interval (Second-Third values). The fourth value in each cell reports the standardized hazard ratio, reflecting the risk associated with a one–standard deviation increases and allowing comparison across measures. The final value in each cell is the adjusted p-value. All models were adjusted for age, sex, and education.

Abbreviations: APOE4= ε4 allele of Apolipoprotein E; AMYL (SUVR)= Standardized Uptake Value Ratio of amyloid-β; HIP = Hippocampus Volume; CFI=Cognitive Function Index; CCB=Cogstate Computerized Battery; AIC=Akaike Information Criterion
